# Supplementary material for: The impact of persistent bacterial bronchitis on the pulmonary microbiome of children
Source: PLoS One. 2017 Dec 27;12(12):e0190075. doi: 10.1371/journal.pone.0190075 (PMC5744971; doi:10.1371/journal.pone.0190075)
Supplement: S1 Appendix — This file includes supplementary methods and tables. (DOCX) [file pone.0190075.s001.docx]

**ONLINE DATA SUPPLEMENT**

Vanessa Craven^1*^, Leah Cuthbertson^2,3*^, Lynne Bingle^1^, William O.C.M. Cookson ^ǂ2,3^, Mark L. Everard^ǂ4^, Miriam F. Moffatt^ǂ2^

**S1 Appendix. Supplementary information**

***Sample collection***

Healthy controls did not have any chronic respiratory symptoms (cough, wheeze, shortness of breath) or a diagnosis of any respiratory conditions. All case subjects had probable or confirmed PBB as diagnosed by a respiratory paediatrician. Demographic data and medical history including respiratory symptoms, atopy, antibiotic therapy, infant feeding methods and familial smoking were recorded (See Table 1). All participants were asked to document symptoms for 7 days post procedure and to return this to the study team.

Immediately after intubation with an ET tube, an Olympus 1.2mm cytology brush was inserted into the ET tube until light resistance was felt at which point the brush was moved up and down 3 times and then removed (blind brush). The tip of the brush was cut into a sterile container using sterile wire cutters and the container immediately put into a box with ice packs and stored at -80°C. In subjects with PBB, after the first brushing the bronchoscope was inserted into the right main bronchus, or the region of most obvious pathology, and a second brushing was taken under direct visualisation (non-blind brush). Prior to use a sterile brush was inserted into the bronchoscope of a selection of patients, the brush was cut into a sterile container using sterile wire cutters. These samples were stored and processed in line with samples as bronchoscopy controls.

***DNA extraction***

DNA was extracted from brushes and swabs using the MPBio FastDNA SPIN Kit for Soil Components. Each sample was resuspended in 978μl Sodium Phosphate Buffer in a Lysing Matrix E tube. One hundred and 22μl of MT buffer was added to each sample (brush or swab) prior to homogenisation using the Precellys-24 at 6,000-2x30-005 for 2 minutes. Tubes were then centrifuged at 14,000 rpm for 10 minutes to pellet the debris. The supernatant was transferred into a sterile 1.5ml microcentrifuge tube.

When extracting from swabs, after the initial homogenisation and centrifugation step, the swab was aseptically transferred into a sterile spin basket in a 1.5ml microcentrifuge tube and centrifuged again at 14,000 rpm for 1 minute to remove any excess fluid. The excess fluid was combined with the supernatant obtained from the initial homogenization and centrifugation.

Next, each sample’s supernatant was combined with 250μl Protein Precipitation Solution and the tube inverted 10 times then centrifuged at 14,000 rpm for 5 minutes. The supernatant was removed and added to a 15ml falcon tube containing 1ml Binding Matrix suspension. This was placed on a rotator platform for 20 minutes then left to settle for 3 minutes before 800μl of the clear supernatant was discarded.

The binding matrix was resuspended in the remaining liquid and 600μl transferred to a spin tube prior to spinning at 14,000 rpm for 1 minute. This was repeated three times until the remaining binding matrix had been through the spin column. The pellet was washed with 500μl of SEWS-M solution by gently resuspending the pellet then spinning at 14,000 rpm for 1 minute. The flow through was discarded and the tube was spun for a further 2 minutes after which it was left to air dry for 1 minute. The binding matrix was then resuspended in 100μl of low EDTA TE buffer and incubated at 55°C in a heat block for 5 minutes. The tube was then centrifuged at 14,000 rpm for 1 minute and the eluted DNA recovered and the stored at -80°C until required.

***Quantitative PCR***

Quantitative PCR (qPCR) was performed using the ViiA 7 Real-time PCR system (Life Technologies, Paisley, UK) and the SYBR Fast qPCR Master mix (KAPA Biosystems, Wilmington, MA, USA). A 253bp product was generated using the primers: 520F, 5’- AYTGGGYDTAAAGNG and 820R, 5’-TACNVGGGTATCTAATCC[1]. All reactions were performed in triplicate and included standards and non-template controls. Standards were generated from near full length cloned 16S rRNA gene of *Vibrio natregens*. Plasmids quantified using Quantit picogreen dsDNA Assay kit (Promega, Madison, USA), samples were then serially diluted 10 fold to form standards ranging from 1 x 10^8^ -1 x 10^4^. Reactions consisted of 7.5μl of SYBR Fast qPCR Master mix, 0.3μl of 10μM dilutions primers and 1.9μl of nuclease free PCR water (CAMBIO). Template DNA was diluted 1:5 and 5μl was added to each reaction. Cycling conditions were: 90°C for 3 minutes followed by 40 cycles of: 95°C for 20 seconds, 50°C for 30 seconds, and 72°C for 30 seconds. Melt curves were run as default from 60 to 95°C, over 15 minutes. Copy numbers per μl of each sample were extrapolated from the cycle threshold using the ViiA7 software (See Table S3).

***DNA sequencing***

PCR amplification was carried out in quadruplicate using; 12.5μl Q5 High-Fidelity PCR master mix (New England Biolabs, Ipswich, Massachusetts, USA), 5μl of each 1.5μM barcoded primer, 1.5μl of nuclease free PCR water (MOBIO, Carlsbad, CA, USA) and 1μl of either sample DNA, extraction control, mock community or nuclease free PCR water. A mock community, comprising of 34 known bacterial species in equal proportion, was included as a sequencing positive control in each sequencing run. The community included both known respiratory bacteria as well as those not expected to be present within the lung. Cycling conditions used for amplification were: 95°C for 2 minutes followed by 35 cycles of 95°C for 20 seconds, 50°C for 20 seconds and 72°C for 5 minutes.

The mock community and negative control products were visualised on a 1.2% agarose gel, to ensure the correct amplicon size and confirm that no contamination was present in the PCR negative control. Replicate PCRs were then pooled and a selection of PCR products from samples were run to ensure an amplicon of the correct size had been generated.

Agencourt™ Ampure beads (Beckman Coulter, High Wycombe, UK) in a ratio of 0.7:1 were added to a uniform volume of each PCR product and incubated at room temperature for 5 minutes. The plate was then placed on a magnetic rack until the beads had been fully captured after which the supernatant was carefully removed by pipette. The beads were washed twice with 100μl of 80% ethanol. Beads were dried at room temperature for 5 minute before they were re-suspended in 30μl of low EDTA, TE buffer (Invitrogen, Paisley, UK). Ampure purified amplicons were quantified using the Quant-iT PicoGreen dsDNA Assay Kit (Life Technologies, Paisley, UK) according to manufacturer’s instructions. Calculated sample concentrations were used to make a single equi-molar pooled sample. Due to the low concentration of the negative control a standard volume of 5μl was added to the equi-molar pool.

The pooled samples were purified and concentrated into 30μl of low EDTA TE buffer using Ampure beads as above. The pooled library was gel purified using the QIAquick Gel Extraction Kit (QIAGEN, Manchester, UK) following manufacturer’s instructions. Recommended extra washes through the spin column were included to improve yield and the library was finally suspended in 30μl of elution buffer.

Prior to sequencing on the Illumina MiSeq, the library was quantified using the rox low Illumina library quantification kit (KAPA Biosystems, London, UK). The pooled library was serially diluted; 1:1,000, 1:2,000, 1:4,000, 1:8,000, to allow accurate quantification in line with the provided standards. The amplicon size was confirmed using the 2100 Bioanalyzer (Agilent Technologies, Santa Clara, CA), as per manufacturer’s instructions, to ensure accurate quantification of the library for sequencing.

The library was loaded into the IlluminaV2 500 cycle (Illumina, Saffron Walden, UK) sequencing cartridge at a concentration of 8pM with a 20% PhiX spike.

***Sequence processing***

Sequences were quality trimmed to 200bp and joined with a maximum of 10% mismatch and a minimum of 150 base pair overlap, prior to de-multiplexing. Open reference UCLUST OTU picking[2] was then used to cluster sequences to 97% similarity using the Silva reference database (www.arb-silva.de), before picking representative sequences. Sequences were then aligned using PyNAST [3] to implement the nearest alignment space termination (NAST) algorithm[4].

Next chimeric sequences were identified and removed using Chimera slayer (http://microbiomeutil.sourceforge.net/). Taxonomic identification was applied using the Ribosomal Database Project (RDP) naive Bayesian classifier[5] retained with the Silva 115 NR database. An OTU table was then created for downstream analysis.

***Contamination controls***

Common contaminants and OTUs with unclassified kingdom were removed prior to further analysis (Table S4). In order to control for contamination steps were taken to account for contamination at each stage of the sequencing process. All samples including the controls were randomised prior to DNA extraction. Extracted samples were similarly randomised for subsequent qPCR after which samples were ordered from highest to lowest qPCR values and sequenced on two MiSeq runs to minimise sequence failure

Post sequencing and OTU picking, OTUs that were found to increase with decreasing qPCR values were removed from further analysis.

Differences between the plates were investigated by the removal of OTUs associated with extraction and PCR negative controls. Adonis was used to investigate if there was a significant plate effect (*p*=<0.001, R^2^=0.028). Despite the significant result the R^2^ value is low. Removal of all OTUs associated with negative controls resulted in a reduction of R^2^=0.016, however the significant plate effect remained. As a result, OTUs were not removed from analysis, however the effect of plate was accounted for in further tests.

**REFERENCES**

1. Lane DJ. Nucleic Acid Techniques in Bacterial Systematics. In: Stackebrandt E GM, editor. Chichester, UK: Wiley; 1991.

2. Edgar RC. Search and clustering orders of magnitude faster than BLAST. *Bioinformatics* 2010;26:2460–2461.  doi: 10.1093/bioinformatics/btq461

3. Caporaso JG, Bittinger K, Bushman FD, DeSantis TZ, Andersen GL, Knight R. PyNAST: a flexible tool for aligning sequences to a template alignment. *Bioinformatics* 2010;26:266–267. doi: 10.1093/bioinformatics/btp636

4. DeSantis TZ, Hugenholtz P, Keller K, Brodie EL, Larsen N, Piceno YM, Phan R, Andersen GL. NAST: a multiple sequence alignment server for comparative analysis of 16S rRNA genes. Nucleic Acids Res 2006;34:W394–399. doi: 10.1093/nar/gkl244

5. Wang X, Mair R, Hatcher C, Theodore MJ, Edmond K, Wu HM, Harcourt BH, Carvalho M da GS, Pimenta F, Nymadawa P, Altantsetseg D, Kirsch M, Satola SW, Cohn A, Messonnier NE, Mayer LW. Detection of bacterial pathogens in Mongolia meningitis surveillance with a new real-time PCR assay to detect *Haemophilus influenzae*. *Int J Med Microbiol* 2011;301:303–309. doi: 10.1016/j.ijmm.2010.11.004

S1 Table. Number of samples sequenced, including controls and repeats.

|  | Number of samples |
| --- | --- |
| Cough Patients samples | 50 |
| Control patient samples | 42 |
| Mother samples | 34 |
| Mock communities | 3 |
| PCR Negative controls | 4 |
| Extraction Controls | 2 |
| Bronchoscope Controls | 11 |
| Total samples sequenced | 146 |

S2 Table. Demographics of mothers sampled.

|  | Count |
| --- | --- |
| Mother | 16 |
| Female | 16 |
| Nose Swab | 16 |
| Throat Swab | 16 |
| Mother of case | 11 |
| Mother of control | 5 |
| Smoke | 0 |
| Breastfed | 8 |

Table S3. Sample information and barcodes.

| Code | | BarcodeSequence | qPCR (ng/µl) | Sample type | Disease | Family |
| --- | --- | --- | --- | --- | --- | --- |
| BB29 | CGAGGCTGTATCCTCT | | 44269.75 | blind brush | Cough | 29 |
| BB7 | AAGAGGCAAAGGAGTA | | 571169.22 | blind brush | Cough | 7 |
| BB2.16 | TCCTGAGCAGAGTAGA | | 14084.21 | blind brush | Control | 15 |
| 9BB2 | CGTACTAGCTAAGCCT | | 21083.04 | blind brush | Control | 9 |
| BB1.20 | CTCTCTACAAGGAGTA | | 84305.83 | blind brush | Control | 19 |
| BB3 | CTCTCTACGTAAGGAG | | 40575.67 | blind brush | Cough | 3 |
| 12BB1 | GCTACGCTTATCCTCT | | 214906.16 | blind brush | Control | 12 |
| BB2.20 | CGTACTAGAAGGAGTA | | 603084.31 | blind brush | Control | 19 |
| BB.32 | GTAGAGGAAAGGAGTA | | 2670724.92 | blind brush | Cough | 32 |
| MN34 | TAGGCATGAGAGTAGA | | 188.71 | nose swab | Mother | 34 |
| BB1.14 | TAGGCATGCTAAGCCT | | Not Detected | blind brush | Cough | 14 |
| BB39 | TAGGCATGAAGGAGTA | | 90312.63 | blind brush | Cough | 39 |
| 36NBB | CGTACTAGGTAAGGAG | | 158025.79 | non-blind brush | Cough | 36 |
| BB28 | CAGAGAGGACTGCATA | | 72306.55 | blind brush | Cough | 28 |
| 21BB | AGGCAGAAGTAAGGAG | | 29219.31 | blind brush | Cough | 21 |
| BB1.27 | GCTACGCTCTCTCTAT | | 64525.98 | blind brush | Control | 27 |
| BB1.16 | GTAGAGGAAGAGTAGA | | 852267.69 | blind brush | Control | 15 |
| 11BB | TCCTGAGCGTAAGGAG | | 1334.62 | blind brush | Cough | 11 |
| NBB26 | CGAGGCTGGTAAGGAG | | 14636.99 | non-blind brush | Cough | 26 |
| BB36 | TAGGCATGCTCTCTAT | | 33049.81 | blind brush | Cough | 36 |
| B2.27 | TAAGGCGATATCCTCT | | 23257.67 | blind brush | Control | 27 |
| 6BB | CTCTCTACCTCTCTAT | | 365285.34 | blind brush | Cough | 6 |
| 8BB | AAGAGGCAGTAAGGAG | | 69200.09 | blind brush | Cough | 8 |
| BB1.16 | CAGAGAGGAAGGAGTA | | 50470.87 | blind brush | Cough | 1 |
| 17NBB | AGGCAGAAGTAAGGAG | | 10683.78 | non-blind brush | Cough | 17 |
| MT39 | TAGGCATGGTAAGGAG | | 82558.06 | throat swab | Mother | 39 |
| 1MN | TCCTGAGCCTCTCTAT | | Not Detected | nose swab | Mother | 1 |
| 20BB2 | CGAGGCTGAAGGAGTA | | 86748.81 | blind brush | Control | 20 |
| 20BB2 | CGAGGCTGCTAAGCCT | | 86748.81 | blind brush | Control | 20 |
| 22BB1 | GGACTCCTGTAAGGAG | | 33729.39 | blind brush | Control | 22 |
| MT29 | TAAGGCGAACTGCATA | | 111843.72 | throat swab | Mother | 29 |
| 24BB1 | TAAGGCGAAAGGAGTA | | 17314.76 | blind brush | Control | 24 |
| 24BB1 | TAAGGCGACTAAGCCT | | 17314.76 | blind brush | Control | 24 |
| 24BB2 | GGACTCCTAGAGTAGA | | 2647.61 | blind brush | Control | 24 |
| 24MT | GGACTCCTAAGGAGTA | | 21299.04 | throat swab | Mother | 24 |
| 24MT | GGACTCCTCTAAGCCT | | 21299.04 | throat swab | Mother | 24 |
| MT27 | CAGAGAGGAGAGTAGA | | 54180.43 | throat swab | Mother | 27 |
| 7NBB | GGACTCCTACTGCATA | | 120566.03 | non-blind brush | Cough | 7 |
| 1MT | GGACTCCTCTAAGCCT | | Not Detected | throat swab | Mother | 1 |
| 33NBB | GCTACGCTCTCTCTAT | | 171.76 | non-blind brush | Cough | 33 |
| 2NBB | CGAGGCTGAGAGTAGA | | 85.13 | non-blind brush | Cough | 2 |
| MT3 | AGGCAGAAACTGCATA | | 116093.98 | throat swab | Mother | 3 |
| 3MN | AAGAGGCATATCCTCT | | 263.57 | nose swab | Mother | 3 |
| MT37 | TCCTGAGCAAGGAGTA | | 229396.82 | throat swab | Mother | 37 |
| 45BB1 | CGTACTAGAAGGAGTA | | 13225.69 | blind brush | Control | 45 |
| 45BB1 | CGTACTAGCTAAGCCT | | 13225.69 | blind brush | Control | 45 |
| 14MT | CTCTCTACCTAAGCCT | | 5576416.50 | throat swab | Mother | 14 |
| BB1.31 | TAGGCATGTATCCTCT | | 38617.24 | blind brush | Control | 31 |
| 4BB | GCTACGCTAAGGAGTA | | 24129.00 | blind brush | Cough | 4 |
| 4BB | GCTACGCTCTAAGCCT | | 24129.00 | blind brush | Cough | 4 |
| 3NBB | AAGAGGCACTCTCTAT | | 31862.25 | non-blind brush | Cough | 3 |
| MT18 | CAGAGAGGCTAAGCCT | | 6585404.25 | throat swab | Mother | 18 |
| MN37 | TCCTGAGCCTCTCTAT | | 23231.57 | nose swab | Mother | 37 |
| 5MT | GCTACGCTCTAAGCCT | | 12338944.33 | throat swab | Mother | 5 |
| NBB39 | GCTACGCTACTGCATA | | 173631.26 | non-blind brush | Cough | 39 |
| BB2.18 | CAGAGAGGGTAAGGAG | | 84377.22 | blind brush | Control | 18 |
| 8NBB | TCCTGAGCTATCCTCT | | 35614.72 | non-blind brush | Cough | 8 |
| NBB21 | GCTACGCTAGAGTAGA | | 55320.18 | non-blind brush | Cough | 21 |
| NBB30 | AGGCAGAACTAAGCCT | | 1022647.58 | non-blind brush | Cough | 30 |
| 5BB | CGAGGCTGAGAGTAGA | | 6737.04 | blind brush | Cough | 5 |
| 5MN | GCTACGCTAGAGTAGA | | 5349.36 | nose swab | Mother | 5 |
| MN39 | TAGGCATGACTGCATA | | 141138.61 | nose swab | Mother | 39 |
| NBB37 | GGACTCCTAAGGAGTA | | 242706.25 | non-blind brush | Cough | 37 |
| MN36 | CGAGGCTGACTGCATA | | 178538.02 | nose swab | Mother | 36 |
| BB43 | CGTACTAGACTGCATA | | 114470.63 | blind brush | Cough | 43 |
| 5NBB | CAGAGAGGTATCCTCT | | 39387.63 | non-blind brush | Cough | 5 |
| MT26 | CGAGGCTGCTAAGCCT | | 35172052.00 | throat swab | Mother | 26 |
| 14NBB | TCCTGAGCCTAAGCCT | | 1563485.63 | non-blind brush | Cough | 14 |
| BB1.23 | AGGCAGAAAGAGTAGA | | 148459.05 | blind brush | Control | 23 |
| 17BB | CTCTCTACACTGCATA | | 49747.66 | blind brush | Cough | 17 |
| BB2.40 | GTAGAGGACTCTCTAT | | 32518.57 | blind brush | Control | 40 |
| NBB28 | TCCTGAGCACTGCATA | | 120264.69 | non-blind brush | Cough | 28 |
| BB1.18 | AAGAGGCAACTGCATA | | 189553.15 | blind brush | Control | 18 |
| BB30 | TAAGGCGAAAGGAGTA | | 209900.83 | blind brush | Cough | 30 |
| 35BB1 | GGACTCCTAGAGTAGA | | 50483.40 | blind brush | Control | 35 |
| 35BB2 | CTCTCTACTATCCTCT | | 39330.19 | blind brush | Control | 35 |
| BB1.41 | CGAGGCTGCTCTCTAT | | 31212.82 | blind brush | Control | 41 |
| 23BB2 | CGTACTAGAGAGTAGA | | 49622.47 | blind brush | Control | 23 |
| 2BB | CGAGGCTGAAGGAGTA | | 425378.14 | blind brush | Cough | 2 |
| BB2.25 | GTAGAGGATATCCTCT | | 46354.83 | blind brush | Control | 25 |
| BB1.9 | TAAGGCGACTAAGCCT | | 608105.15 | blind brush | Control | 9 |
| NBB4 | AAGAGGCAAGAGTAGA | | 63943.23 | non-blind brush | Cough | 4 |
| 13BB1 | TAAGGCGAGTAAGGAG | | 66236.11 | blind brush | Control | 13 |
| 6NBB | CAGAGAGGCTCTCTAT | | 26201.02 | non-blind brush | Cough | 6 |
| BB2.42 | GTAGAGGAGTAAGGAG | | 92091.93 | blind brush | Cough | 42 |
| BB1.16 | CTCTCTACAGAGTAGA | | 52756.64 | blind brush | Control | 16 |
| BB238 | TCCTGAGCGTAAGGAG | | 72775.66 | blind brush | Control | 38 |
| NBB43 | GCTACGCTGTAAGGAG | | 51392.32 | non-blind brush | Cough | 43 |
| BB37 | AGGCAGAAAAGGAGTA | | 219282.81 | blind brush | Cough | 37 |
| BB1.25 | AGGCAGAATATCCTCT | | 34209.98 | blind brush | Control | 25 |
| BB2.13 | TAAGGCGAAGAGTAGA | | 46825.40 | blind brush | Control | 13 |
| 20BB1 | GTAGAGGAACTGCATA | | 195068.41 | blind brush | Control | 20 |
| B1.11 | GGACTCCTGTAAGGAG | | 79494.47 | non-blind brush | Cough | 11 |
| 10BB1 | AAGAGGCATATCCTCT | | 44633.87 | blind brush | Control | 10 |
| 1NBB | GCTACGCTAAGGAGTA | | 388412.20 | non-blind brush | Cough | 1 |
| BB33 | GGACTCCTTATCCTCT | | 38613.31 | blind brush | Cough | 33 |
| 9MT | GTAGAGGATATCCTCT | | Not Detected | throat swab | Mother | 9 |
| BB1.38 | TCCTGAGCAAGGAGTA | | 361035.17 | blind brush | Control | 38 |
| BB1.38 | TCCTGAGCCTAAGCCT | | 361035.17 | blind brush | Control | 38 |
| BB1.40 | TAGGCATGAGAGTAGA | | 3446.35 | blind brush | Control | 40 |
| BB1.41 | CAGAGAGGTATCCTCT | | 25782.18 | blind brush | Control | 44 |
| BB2.22 | TAAGGCGAGTAAGGAG | | 129.36 | blind brush | Control | 22 |
| BB231 | AGGCAGAAAAGGAGTA | | 19241.28 | blind brush | Control | 31 |
| BB1.31 | AGGCAGAACTAAGCCT | | 38617.24 | blind brush | Control | 31 |
| BB2.45 | CTCTCTACGTAAGGAG | | 88132.96 | blind brush | Control | 45 |
| BB26 | AGGCAGAAAGAGTAGA | | 6961.20 | blind brush | Cough | 26 |
| BB34 | TCCTGAGCAGAGTAGA | | 319805.36 | blind brush | Cough | 34 |
| CB28 | GTAGAGGACTCTCTAT | | 528.80 | bronchoscopy | Brush control | 28 |
| CB29 | AGGCAGAATATCCTCT | | Not Detected | bronchoscopy | Brush control | 29 |
| CB32 | CTCTCTACCTCTCTAT | | 2526.07 | bronchoscopy | Brush control | 32 |
| CB33 | CAGAGAGGCTCTCTAT | | Not Detected | bronchoscopy | Brush control | 33 |
| CB34 | GCTACGCTTATCCTCT | | Not Detected | bronchoscopy | Brush control | 34 |
| CB36 | CTCTCTACTATCCTCT | | 61296.70 | bronchoscopy | Brush control | 36 |
| CB39 | CGTACTAGTATCCTCT | | Not Detected | bronchoscopy | Brush control | 39 |
| CB42 | TAGGCATGCTCTCTAT | | Not Detected | bronchoscopy | Brush control | 42 |
| CB43 | TCCTGAGCTATCCTCT | | 50.24 | bronchoscopy | Brush control | 43 |
| CB37 | CAGAGAGGAAGGAGTA | | Not Detected | bronchoscopy | Brush control | 37 |
| CB37 | CAGAGAGGCTAAGCCT | | Not Detected | bronchoscopy | Brush control | 37 |
| KC31214 | AAGAGGCACTCTCTAT | | Not Detected | Kit control | Kit control | control |
| KC41214 | TAAGGCGATATCCTCT | | Not Detected | Kit control | Kit control | control |
| MN14 | CGTACTAGAGAGTAGA | | 1563.47 | nose swab | Mother | 14 |
| MN18 | CGTACTAGGTAAGGAG | | 10665.76 | nose swab | Mother | 18 |
| MN2 | AGGCAGAACTCTCTAT | | Not Detected | nose swab | Mother | 2 |
| MN34 | CGAGGCTGTATCCTCT | | 51854.96 | nose swab | Mother | 24 |
| MN25 | GTAGAGGAAGAGTAGA | | 9779.18 | nose swab | Mother | 25 |
| MN26 | TAAGGCGAAGAGTAGA | | 10981.88 | nose swab | Mother | 26 |
| MN27 | CTCTCTACAAGGAGTA | | Not Detected | nose swab | Mother | 27 |
| MN27 | CTCTCTACCTAAGCCT | | Not Detected | nose swab | Mother | 27 |
| MN29 | CGAGGCTGCTCTCTAT | | Not Detected | nose swab | Mother | 29 |
| MN9 | CTCTCTACAGAGTAGA | | 4268.38 | nose swab | Mother | 9 |
| Mock | AAGAGGCACTAAGCCT | | NA | Mock | NA |  |
| Mock | AAGAGGCAAAGGAGTA | | NA | Mock | NA |  |
| Mock | AAGAGGCACTAAGCCT | | NA | Mock | NA |  |
| MT2 | CAGAGAGGAGAGTAGA | | 15506.14 | throat swab | Mother | 2 |
| MT25 | AAGAGGCAAGAGTAGA | | Not Detected | throat swab | Mother | 25 |
| MT34 | GGACTCCTTATCCTCT | | 2206.05 | throat swab | Mother | 34 |
| MT36 | TAGGCATGTATCCTCT | | 2046875.88 | throat swab | Mother | 36 |
| NBB29 | GGACTCCTCTCTCTAT | | Not Detected | non-blind brush | Cough | 29 |
| NBB32 | TAGGCATGAAGGAGTA | | 21358.31 | non-blind brush | Cough | 32 |
| NBB32 | TAGGCATGCTAAGCCT | | 21358.31 | non-blind brush | Cough | 32 |
| NBB34 | CAGAGAGGGTAAGGAG | | 4780.55 | non-blind brush | Cough | 34 |
| NBB42 | TAGGCATGGTAAGGAG | | 13989.94 | non-blind brush | Cough | 42 |
| neg | GTAGAGGAAAGGAGTA | | NA | Negative | NA |  |
| neg | GTAGAGGACTAAGCCT | | NA | Negative | NA |  |
| neg | GTAGAGGAACTGCATA | | NA | Negative | NA |  |
| neg | GTAGAGGACTAAGCCT | | NA | Negative | NA |  |

Table S4. Pre-processing contamination removal

| OTUs removed as a standard | |
| --- | --- |
| Kingdom | Unclassified |
| Phylum | Cyanobacteria |
| Order | Rhodobacterales |
| Order | Rhizobiales |
| Order | Methylophilales |
| Family | Oxalobacteraceae |
| Genus | Derxia |
| Genus | Rhodococcus |
| OTUs removed due to contamination from the Mock | |
| Genus | Vibrio |
| OTUs removed, increasing abundance with decreasing qPCR | |
| OTUID | Stenotrophomonas_4097 |
| OTUID | Delftia_2880 |
| OTUID | Delftia_2881 |

Supplementary Figures

Figure S1. Comparison of community alpha diversity measures between blind and non-blind brushes from PBB patients. Using a Wilcoxon paired sign rank test no significant difference was observed between sampling methods. Richness; Z = 1.843, *P* = 0.068, Shannon-Weiner; Z = -0.017, *P* = 1, Simpsons; Z = 0.261, *P* = 0.812, evenness; Z = -0.052, *P* = 0.973.

Figure S2. Differences in bacterial diversity between PBB patients and healthy controls. Using a Wilcoxon sign rank test significant differences in richness (W = 90.5, *P* = 0.001), Shannon-Weiner (W = 70, *P* < 0.001), Simpson’s reciprocal (W = 76, *P* < 0.001) and evenness (W = 65, *P* < 0.001) were observed between the two groups.

Figure S3. Volcano plot indicating the OTUs significantly increased in PBB patients (*P* < 0.001). Grey points indicate OTUs with *P* > 0.001. Colours indicate OTU genus, while size indicates the sum of the reads in each OTU. Significant OTUs showed a more than 2.5 fold increase in abundance compared to controls.

Figure S4. Ordered bar chart of the top 20 OTUs present in PBB patients with and without a diagnosis of wheeze. Samples are ordered by a Bray Curtis dissimilarity hierarchical cluster upper plot with the lower plot indicating if patient has a diagnosis of wheeze. Key to colours used for each genus is included.

Figure S5. NMDS based of Bray-Curtis dissimilarity of the bacterial communities, rarefied to 1154 reads, recovered from both throat and nose swabs of participant mothers. Nose swabs are indicated in blue, throat swabs are indicated in red. Mothers of PBB patients are indicated by triangles while the mothers of controls are shown by circles.

Figure S6. Ordered bar chart of the top 20 OTUs present in both paired throat and nose swabs from mothers. Rarefaction resulted in the removal of some samples due to low sequence numbers, the paired samples from these patients were removed to allow paired analysis. Samples are ordered by a Bray Curtis dissimilarity hierarchical cluster. Key to colours used for each genus is included. Log10 copies of 16S rRNA gene per µl of sample is shown in the lower plot with colour indicating sample type; blue being nasal swab and red being throat swab. Note: missing data 25 (Mother of Patient 25 throat) and 29 (Mother of patient 29 nose) samples had DNA concentrations below the detection limit of the qPCR assay.
