# Supplementary material for: The impact of persistent bacterial bronchitis on the pulmonary microbiome of children
Source: PLoS One. 2017 Dec 27;12(12):e0190075. doi: 10.1371/journal.pone.0190075 (PMC5744971; doi:10.1371/journal.pone.0190075)
Supplement: S1 Table — (DOCX) [file pone.0190075.s002.docx]

S1 Table. Number of samples sequenced, including controls and repeats.

|  | Number of samples |
| --- | --- |
| Cough Patients samples | 50 |
| Control patient samples | 42 |
| Mother samples | 34 |
| Mock communities | 3 |
| PCR Negative controls | 4 |
| Extraction Controls | 2 |
| Bronchoscope Controls | 11 |
| Total samples sequenced | 146 |
