# Supplementary material for: The impact of persistent bacterial bronchitis on the pulmonary microbiome of children
Source: PLoS One. 2017 Dec 27;12(12):e0190075. doi: 10.1371/journal.pone.0190075 (PMC5744971; doi:10.1371/journal.pone.0190075)
Supplement: S2 Table — (DOCX) [file pone.0190075.s003.docx]

S2 Table. Demographics of mothers sampled.

|  | Count |
| --- | --- |
| Mother | 16 |
| Female | 16 |
| Nose Swab | 16 |
| Throat Swab | 16 |
| Mother of case | 11 |
| Mother of control | 5 |
| Smoke | 0 |
| Breastfed | 8 |
