# Supplementary figures and images for: The impact of persistent bacterial bronchitis on the pulmonary microbiome of children
Source: PLoS One. 2017 Dec 27;12(12):e0190075. doi: 10.1371/journal.pone.0190075 (PMC5744971; doi:10.1371/journal.pone.0190075)

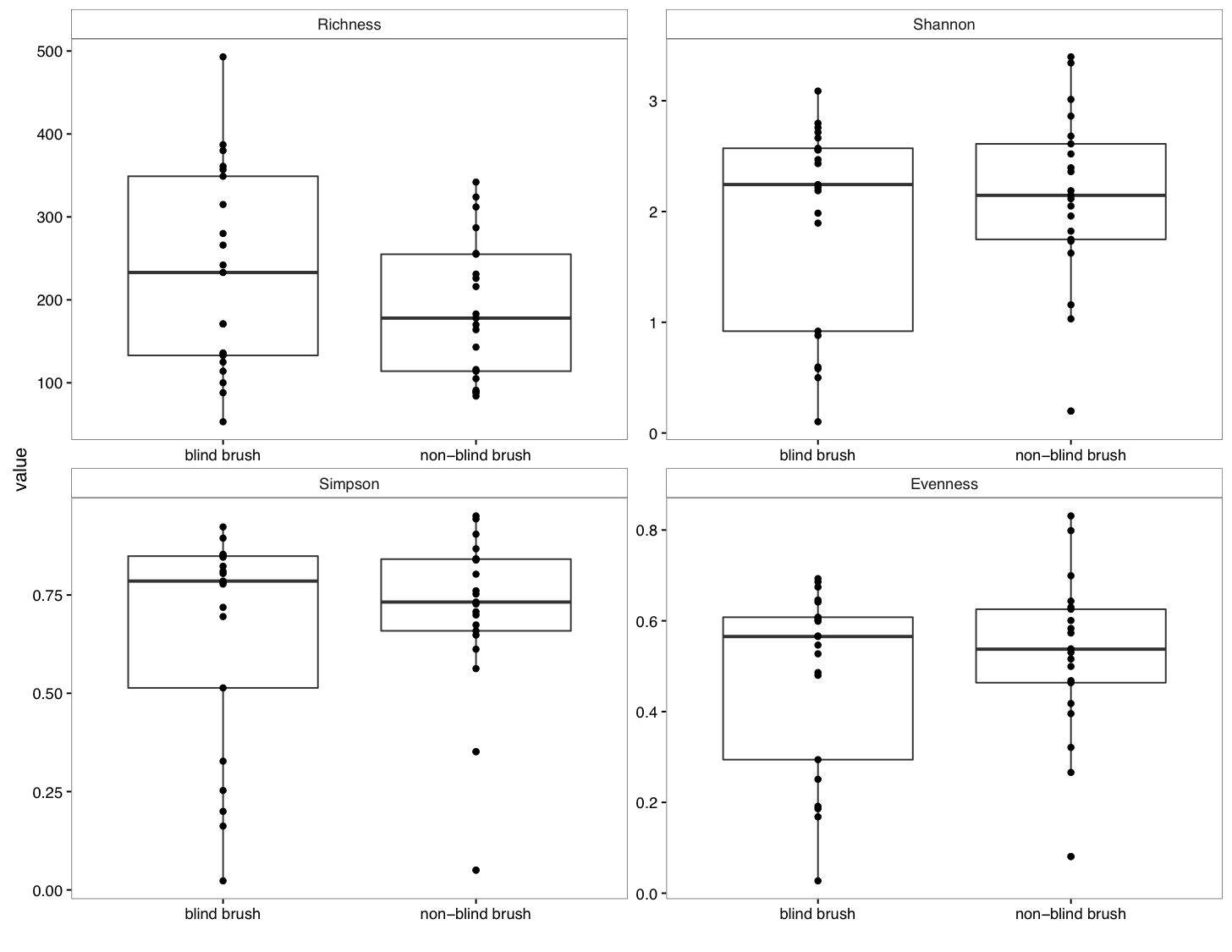

Supplement: S1 Fig — Using a Wilcoxon paired sign rank test no significant difference was observed between sampling methods. Richness; Z = 1.843, P = 0.068, Shannon-Weiner; Z = -0.017, P = 1, Simpsons; Z = 0.261, P = 0.812, evenness; Z = -0.052, P = 0.973. (TIF) [file pone.0190075.s004.tif]

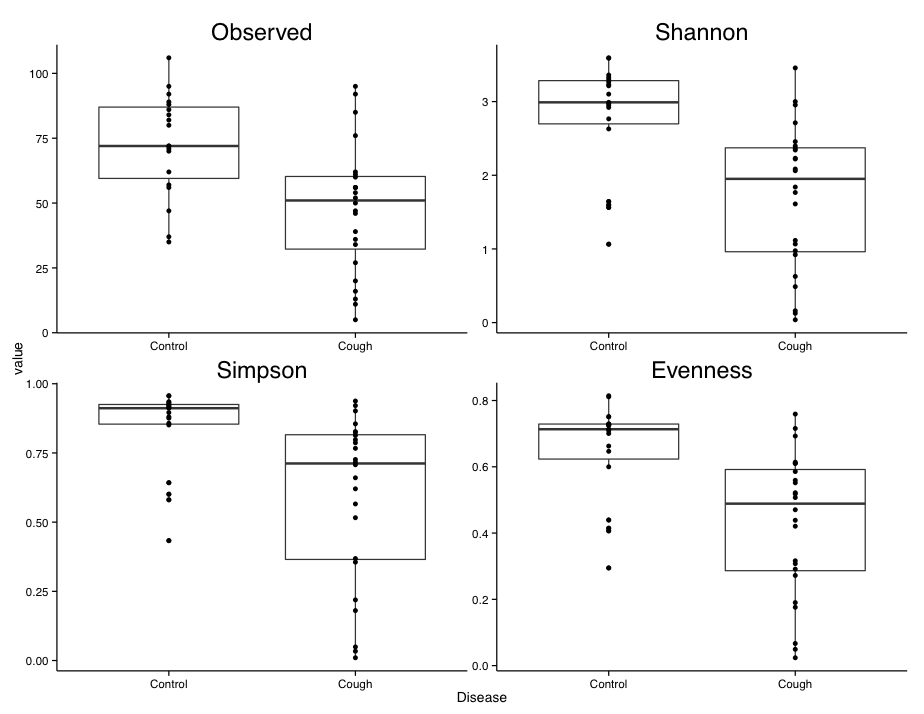

Supplement: S2 Fig — Using a Wilcoxon sign rank test significant differences in richness (W = 90.5, P = 0.001), Shannon-Weiner (W = 70, P < 0.001), Simpson’s reciprocal (W = 76, P < 0.001) and evenness (W = 65, P < 0.001) were observed between the two groups. (TIF) [file pone.0190075.s005.tif]

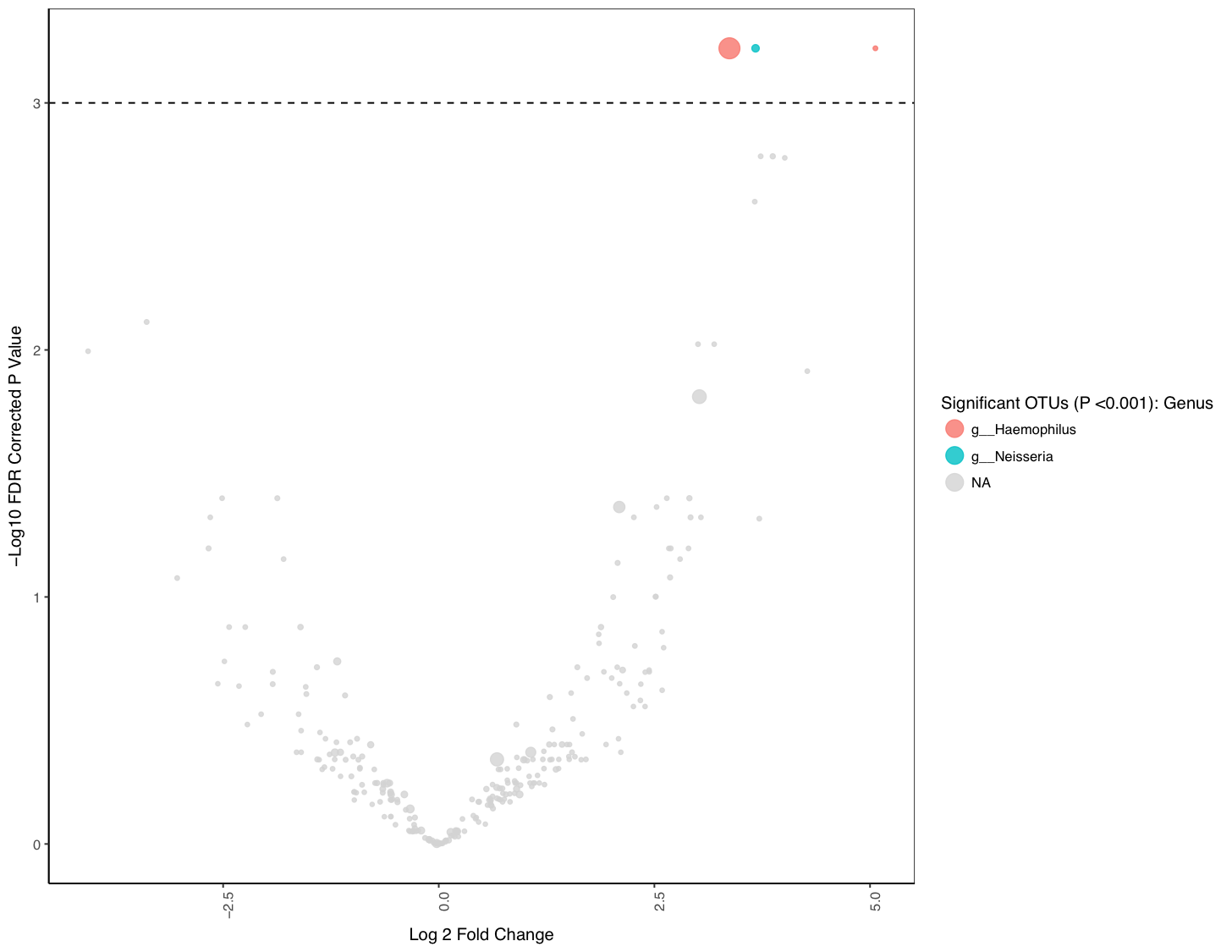

Supplement: S3 Fig — Grey points indicate OTUs with P > 0.001. Colours indicate OTU genus, while size indicates the sum of the reads in each OTU. Significant OTUs showed a more than 2.5 fold increase in abundance compared to controls. (TIF) [file pone.0190075.s006.tif]

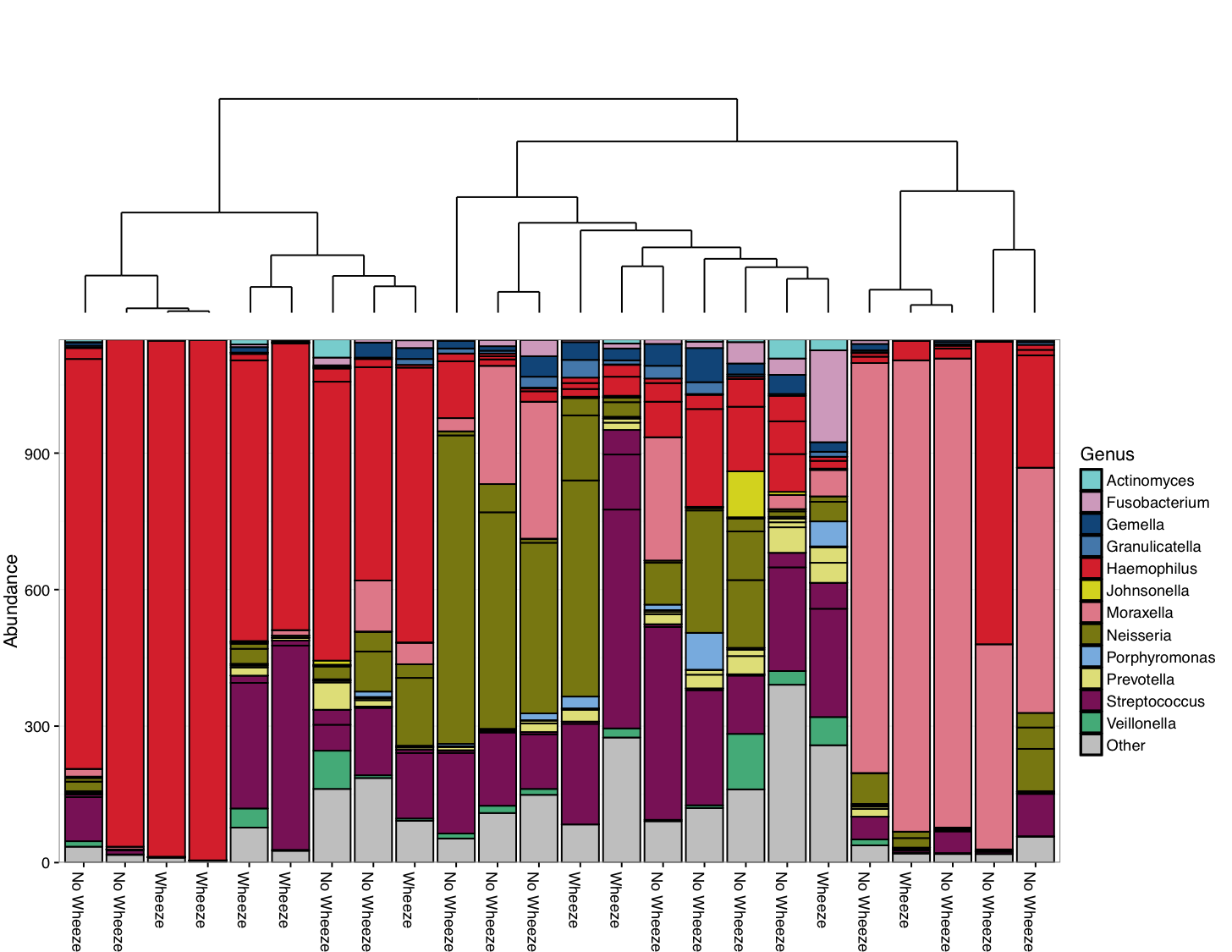

Supplement: S4 Fig — Samples are ordered by a Bray Curtis dissimilarity hierarchical cluster upper plot with the lower plot indicating if patient has a diagnosis of wheeze. Key to colours used for each genus is included. (TIF) [file pone.0190075.s007.tif]

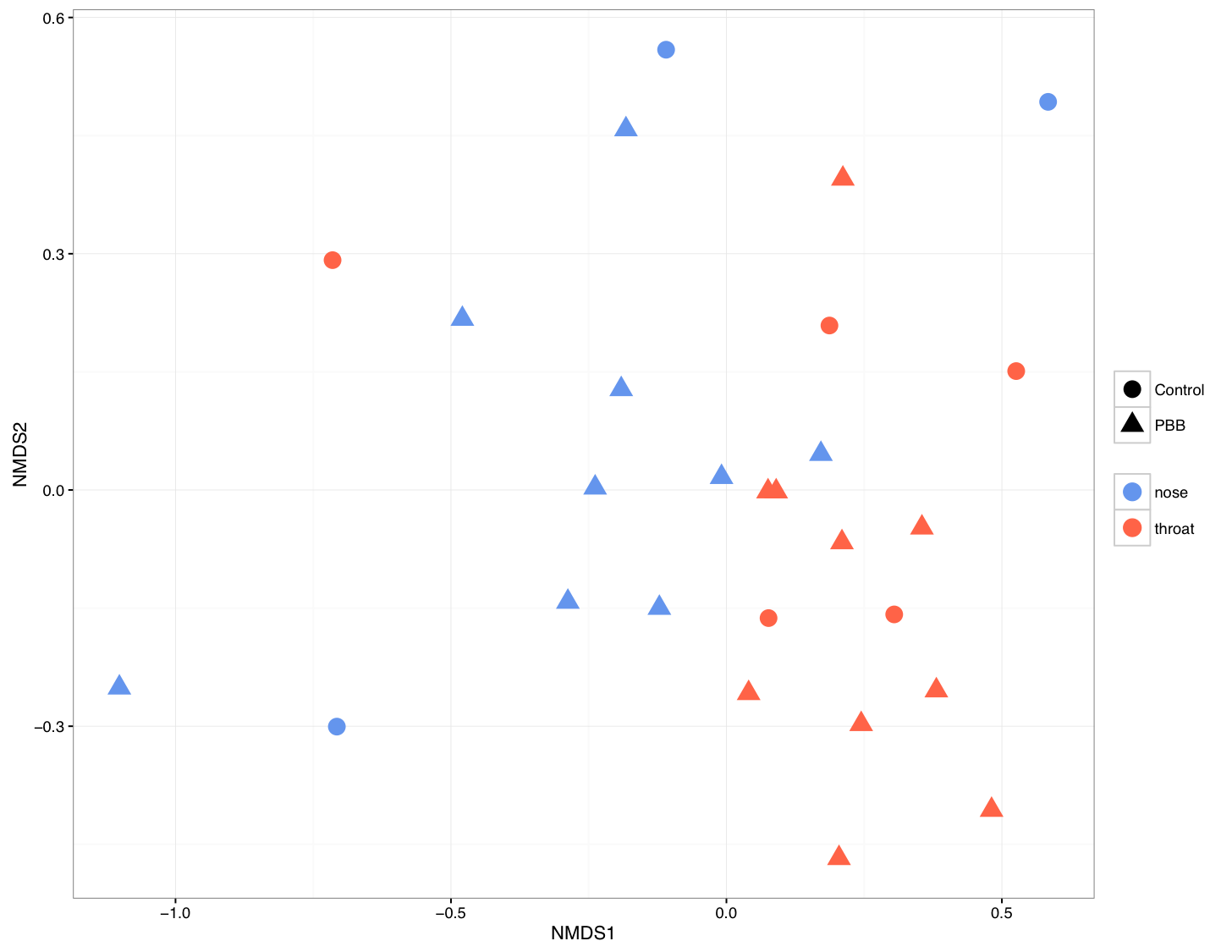

Supplement: S5 Fig — Nose swabs are indicated in blue, throat swabs are indicated in red. Mothers of PBB patients are indicated by triangles while the mothers of controls are shown by circles. (TIF) [file pone.0190075.s008.tif]

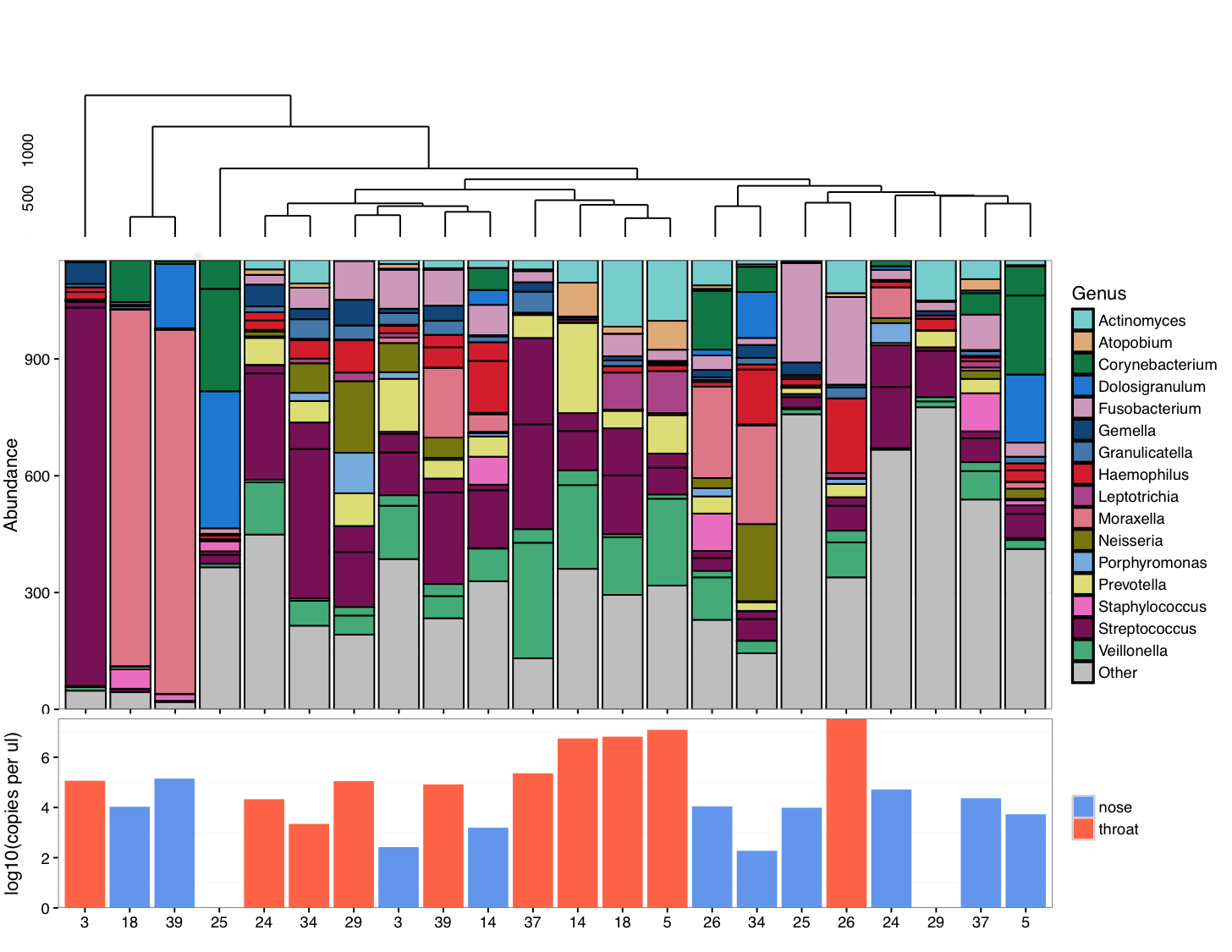

Supplement: S6 Fig — Rarefaction resulted in the removal of some samples due to low sequence numbers, the paired samples from these patients were removed to allow paired analysis. Samples are ordered by a Bray Curtis dissimilarity hierarchical cluster. Key to colours used for each genus is included. Log10 copies of 16S rRNA gene per μl of sample is shown in the lower plot with colour indicating sample type; blue being nasal swab and red being throat swab. Note: missing data 25 (Mother of Patient 25 throat) and 29 (Mother of patient 29 nose) samples had DNA concentrations below the detection limit of the qPCR assay. (TIF) [file pone.0190075.s009.tif]
